# Identification and Characterization of Peripheral T-Cell Lymphoma-Associated SEREX Antigens

**DOI:** 10.1371/journal.pone.0023916

**Published:** 2011-08-22

**Authors:** Christopher D. O. Cooper, Charles H. Lawrie, Amanda P. Liggins, Graham P. Collins, Christian S. R. Hatton, Karen Pulford, Alison H. Banham

**Affiliations:** 1 Nuffield Department of Clinical Laboratory Sciences, Oxford NIHR Biomedical Research Centre, University of Oxford, John Radcliffe Hospital, Oxford, United Kingdom; 2 Structural Genomics Consortium, University of Oxford, Oxford, United Kingdom; 3 Biodonostia Institute, San Sebastián, Spain; 4 Department of Haematology, John Radcliffe Hospital, Oxford, United Kingdom; Univesity of Texas Southwestern Medical Center at Dallas, United States of America

## Abstract

Peripheral T-cell lymphomas (PTCL) are generally less common and pursue a more aggressive clinical course than B-cell lymphomas, with the T-cell phenotype itself being a poor prognostic factor in adult non-Hodgkin lymphoma (NHL). With notable exceptions such as ALK^+^ anaplastic large cell lymphoma (ALCL, ALK+), the molecular abnormalities in PTCL remain poorly characterised. We had previously identified circulating antibodies to ALK in patients with ALCL, ALK^+^. Thus, as a strategy to identify potential antigens associated with the pathogenesis of PTCL, not otherwise specified (PTCL, NOS), we screened a testis cDNA library with sera from four PTCL, NOS patients using the SEREX (serological analysis of recombinant cDNA expression libraries) technique. We identified nine PTCL, NOS-associated antigens whose immunological reactivity was further investigated using sera from 52 B- and T-cell lymphoma patients and 17 normal controls. The centrosomal protein CEP250 was specifically recognised by patients sera and showed increased protein expression in cell lines derived from T-cell versus B-cell malignancies. TCEB3, BECN1, and two previously uncharacterised proteins, c14orf93 and ZBTB44, were preferentially recognised by patients' sera. Transcripts for all nine genes were identified in 39 cancer cell lines and the five genes encoding preferentially lymphoma-recognised antigens were widely expressed in normal tissues and mononuclear cell subsets. In summary, this study identifies novel molecules that are immunologically recognised *in vivo* by patients with PTCL, NOS. Future studies are needed to determine whether these tumor antigens play a role in the pathogenesis of PTCL.

## Introduction

Peripheral T-cell lymphomas (PTCL) are relatively uncommon, and together with NK-cell neoplasms, comprise approximately 12% of all non-Hodgkin lymphomas (NHL) [1]. Because of the lack of consistent immunophenotypic and genetic markers, approximately 30% of these tumours are classified into a category described as “not otherwise specified” (PTCL, NOS) in the current World Health Organisation classification scheme [2]. In Western countries nodal tumours are the most common form and include three main subtypes: PTCL, NOS, angioimmunoblastic T-cell lymphoma (AITL), and anaplastic large cell lymphoma (ALCL), anaplastic lymphoma kinase (ALK)-positive (ALCL, ALK+) [3]. PTCL generally express T-cell associated markers, with nodal tumours most often being CD4^+^ and extranodal cases being CD8^+^. However, in approximately half of cases these two antigens are either both absent or are co-expressed [4].

The molecular abnormalities underlying PTCL are generally poorly understood, and in contrast to data from patients with B-NHL, reports are relatively rare. While complex and recurrent cytogenetic abnormalities have been described, specific genetic alterations remain elusive and have not been correlated with histologic subgroup or clinical outcome [5]. The notable exception is ALCL, ALK+ which express a variety of oncogenic ALK fusion proteins [6], [7] and are routinely identified as a specific entity using an anti-ALK monoclonal antibody (ALK1) [8].

Gene expression profiling has made an important contribution to the molecular classification of distinct disease entities and subtypes within B-cell NHL, providing information that impacts on both diagnosis and treatment (reviewed by [9]). Initial expression profiling studies of nodal PTCL revealed differences between T-cell lymphomas and normal T lymphocytes and also molecular heterogeneity, particularly in PTCL, NOS. It was, however, difficult to separate ALCL, ALK+, AITL and PTCL, NOS, and to assess the contribution of reactive cells within the tumour microenvironment [10]. More recently this approach has suggested that AITL may be derived from follicular helper T cells that are normally present in germinal centres and that a subset of CD30-negative PTCL, NOS may derive from or be related to AITL [11], [12]; while the remaining PTCL, NOS are most closely related to activated peripheral T lymphocytes [13]. More recently, the proliferation signature has been identified as being of importance in nodal PTCL [14] and new potential therapeutic targets, such as PDGFRα, have been identified [13].

As a group, PTCL are clinically aggressive and have a worse prognosis than B-cell lymphomas when treated with chemotherapy. Notably the T-cell immunophenotype itself is regarded as a poor prognostic factor in studies of NHL [15]. This poor response to therapy confers a correspondingly shorter 5-year overall survival rate, which can be as low as 21% for patients with a high-to-intermediate IPI (International Prognostic Index) score or 6% for those with high-risk disease [16]. An exception to this general rule is ALCL, ALK+ in which the majority of patients have a relatively good prognosis [17]. Because of the dismal prognosis in PTCL, NOS there are a variety of new experimental approaches to treatment being investigated [18] and data suggest that high-dose chemotherapy and autologous stem cell transplantation as a frontline consolidation therapy may improve treatment outcome [19].

The future development of immunotherapeutic options may also help to improve the outcome for PTCL patients. Immunotherapeutic approaches using monoclonal antibodies such as Alemtuzumab (anti-CD52) and Ontak (denileukin diftitox, anti-CD25) are already showing promise in these malignancies [18]. Lymphoma vaccination may also have a role in the treatment of PTCL. However, this approach requires both the identification and characterisation of antigenic tumour proteins. We have already demonstrated that ALCL, ALK+ patients mount both humoral and cellular immune responses to the ALK protein and that this may be predictive of outcome [20], [21], [22]. Thus circulating antibodies in T-cell lymphoma patients can be raised to molecules that play an essential role in disease pathology. The SEREX technique (serological analysis of recombinant cDNA expression libraries) identifies antigenic tumour-associated molecules that are recognised by naturally occurring circulating antibodies from patients, and has already been successfully used to identify cutaneous T-cell lymphoma-associated antigens [23], [24], [25], [26]. Here we report the identification and further characterisation of nine lymphoma-associated SEREX antigens that are recognised by circulating antibodies in patients with PTCL, NOS.

## Materials and Methods

### Ethics statement

Patient samples were collected in accordance with the Declaration of Helsinki and approved by the local ethics committee (Oxfordshire REC C; Ref no. C02.354). Patients gave written informed consent for the sample collection.

### Patient samples

Patient blood samples were obtained from the Department of Haematology, John Radcliffe Hospital, Oxford, UK. Normal serum samples were obtained from the National Blood Service (Bristol, UK) with appropriate age/sex matching to patient sera where possible. CD19^+^, CD3^+^, CD27^+^, IgD^+^ and IgD^−ve^ peripheral blood cells were purified from pooled buffy coat samples taken from healthy volunteers (n = 12) using immunomagnetic selection (Miltenyi Biotec, Germany) according to the manufacturer's instructions, as previously described [27]. Lymphocyte populations were routinely purified to >90% purity as determined by FACS analysis. Additional resting or activated lymphocyte or monocyte sub-populations and normal tissue cDNA were from the respective Human MTC panels I, II or Human Blood Fractions (BD Biosciences, Oxford, UK).

### Cell culture

Cell lines were maintained in RPMI 1640 medium (Sigma Aldrich, UK) supplemented with 10% foetal calf serum (20% for L540 and OCI-Ly10), 2.1 mM L-Glutamine and antibiotics (penicillin (5000 U/ml) and streptomycin (5000 µg/ml), (Invitrogen, Paisley, UK) in an atmosphere of 5% CO_2_ at 37°C. Cells were washed in RNase-free PBS prior to mRNA or protein extraction.

### Serum preparation

Serum was harvested from total coagulated blood by centrifugation and cleaned, as previously described [28], in order to remove any bacteriophage or bacterial responsive antibodies.

### Immunoscreening of cDNA library

A testis cDNA expression library (Stratagene, La Jolla, USA) was screened using allogeneic sera as previously described [28]. Briefly, *Escherichia coli* XL1-Blue MRF' were infected with the recombinant phage library, plated at a density of 10000–20000 pfu per 140 mm NZY media plate, recombinant protein expression induced with 2 mM isopropyl-β-D-1-thiogalactopyranoside (IPTG) and plates incubated at 37°C overnight. Following protein transfer onto nitrocellulose, membranes were blocked with blocked in 5% (w/v) non-fat milk in TBS/T, (20 mM Tris, 137 mM NaCl, 0.05% Tween-20) for 1 hour and then incubated overnight in 1/100 diluted cleaned serum. After washing in TBS/T membranes were incubated for 1 hour with 1/5000 diluted alkaline phosphatase-conjugated anti-human IgG (Fc fragment specific, Pierce, Tattenhall, UK) or anti-human IgM (Sigma) and reactive plaques were visualised with 5-bromo-4-chloro-3-indoyl phosphate p-toluidene salt (BCIP) and p-nitroblue tetrazolium chloride (NBT). Positively-reacting plaques were excised, phage eluted and re-plated in order for secondary screening with the respective sera to confirm phage reactivity. Positive plaques were again excised and phage eluted. Phage encoding Ig were identified by screening with IgG secondary antibody and discarded. Phage clones were subjected to tertiary screening with multiple sera from lymphoma patients and normal controls to assess the frequency and specificity of their immunological recognition, as described previously [28].

### Sequence analysis of identified antigens

pBK-CMV phagemids were *in vivo* excised from the ZAP Express vector using ExAssist helper phage and the *E. coli* XLOLR host strain according to the manufacturer's instructions (Stratagene) and DNA was purified by miniprep (Qiagen, Crawley, UK). Cloned inserts were sequenced (MWG-Biotech, London, UK) and data analysed using BLAST (http://www.ncbi.nlm.nih.gov), ELM (http://elm.org.eu), PSORT II (http://psort.nibb.ac.jp), InterProScan (http://www.ebi.ac.uk/Tools/InterProScan/) and SMART (http://smart.embl-heidelberg.de).

### RNA extraction and cDNA synthesis

mRNA from cell lines (1×10^7^ cells) was extracted using μMACS mRNA Isolation kits (Miltenyi Biotec, Bergisch Gladbach, Germany) and treated with DNaseI (Ambion, Warrington, UK). Total RNA was isolated from immuno-purified B cells (CD19^+^) and T cells (CD3^+^) with Trizol (Invitrogen) as recommended by the manufacturer and treated with DNaseI. RNA was quantified with a Nanodrop ND-1000 UV spectrophotometer (Nanodrop Technologies, Wilmington, USA). cDNA was synthesised at 50°C for 60 minutes in a 20 µl reaction mix containing 100 ng mRNA or 500 ng total RNA, 200 U Superscript™ III reverse transcriptase, and 1× First strand buffer (Invitrogen), 500 µM dNTPs, 250 ng random hexamers, 5 mM DTT and 1 µl RNasin® ribonuclease inhibitor (Promega, Madison, WI, USA).

### RT-PCR

Oligonucleotide primers (MWG-Biotech) are shown in Table S1. The integrity of cDNA templates was assessed using primers to *TBP* (TATA-box binding protein) and *GAPDH* (glyceraldehyde-3-phosphate dehydrogenase). PCR reactions (20 µl) contained either 0.5 µl of 1/20 diluted cell line cDNA or 0.5 µl of the Human MTC panels I, II and Human Blood Fractions (BD Biosciences, Oxford, UK), 200 µM each dNTP, 1 µM each primer, 1× PCR buffer, 1.5 µM MgCl_2_ and 1 U JumpStart Taq (Sigma, Gillingham, UK). Cycling parameters were as follows: initial denaturation (97°C, 3 min), then 30 cycles of denaturation (95°C, 60 sec), annealing (50°C, 60 sec) and extension (72°C, 60 sec). PCR products were resolved by 2% TBE-agarose gel electrophoresis and cloned with the pGEM®-T Easy system (Promega) and sequenced (MWG-Biotech) to confirm their identity.

### Real-time quantitative PCR (qRT-PCR)

The mRNA transcript levels were measured using pre-designed Taqman® gene expression assays for PTCL, NOS antigens and an endogenous control assay for *TBP* (Table S1) (Applied Biosystems, Foster City, CA, USA). qRT-PCR was performed on a Chromo4 Real-Time PCR detection system (Bio-Rad, Hercules, CA, USA) in a 20 µl reaction volume containing 1 µl of 1/20 diluted cDNA, 10 µl 2× SensiMix reaction buffer (Quantace, London, UK) and 1 µl of the appropriate 20× Taqman assay. Taqman reactions were performed in triplicate and the mean measurement used. PTCL, NOS antigen gene expression data was normalised to the endogenous reference *TBP* [29], [30] to account for differences in RNA quality and reverse transcription efficiency (ΔC_t_ = C_t_^GENE^−C_t_^TBP^), allowing calculation of normalised gene expression (2^−ΔCt^). Normalised gene expression in B-cell or T-cell lines was related to the average ΔCt from a cohort of either 6 normal CD19^+^ B-cell samples or 6 normal CD3^+^ T-cell samples (3 male, 3 female) respectively (ΔΔC_t_ = average ΔC_t_^normals^−ΔC_t_^sample^), thereby allowing calculation of relative gene expression (2^ΔΔCt^).

### Statistical analysis

mRNA expression was compared between cell lines and control normal samples by a two-tailed Mann-Whitney statistical test to 95% confidence, implemented in Prism (GraphPad, San Diego, CA, USA).

### Western blotting

Whole cell lysates from 1×10^7^ cells were prepared in RIPA buffer [31] and normalised by Bradford assay (Sigma) prior to mixing with Laemmli loading buffer [31]. 15 µg of protein extract was resolved using 10% SDS-PAGE and blotted with a semi-dry apparatus (GE Healthcare, Chalfont St Giles, UK) onto PVDF membrane (Millipore, Billerica, MA, USA). Primary antibodies were diluted 1/200 in blocking buffer (5% w/v non-fat dry milk, 1× TBS, 0.1% Tween-20) unless otherwise stated and sourced as follows: Beclin1 (D-18, Santa Cruz, CA, USA), TCEB3 (R-19 & H-300, Santa Cruz), ODF2 (L-20 & N-20, Santa Cruz), RIF1 (ab13422, Abcam, Cambridge, UK), CEP250/c-nap1 (BD Biosciences), BTBD15 (ZBTB44) (1/1000, Aviva Systems Biology, San Diego, CA, USA), and RBPJ (D-20, Santa Cruz). Antigen/antibody complexes were detected using the relevant secondary antibody conjugated to horseradish peroxidase (HRP) (Dako, Glostrup, Denmark) and detected with ECL reagent (GE Healthcare). Western blots were stripped where applicable with Restore western blot stripping buffer (Pierce, Rockford, IL, USA) according to the manufacturer's instructions and re-probed as above using loading control antibodies to GAPDH (1/1000 ab9485, Abcam) or TBP (1/2000, ab818, Abcam).

## Results

### Serological identification and sequence analysis of PTCL, NOS-associated antigens

A testis library was screened using the SEREX technique with pooled sera from four patients with PTCL, NOS to increase the likelihood of identifying cancer testis antigens. The clinical details of the patients whose sera were used for library screening are as described (Table S1). Cancer testis antigens represent particularly attractive candidates for immunotherapy because of their widespread neoplastic expression and restricted normal tissue distribution [32]. Nineteen non-Ig clones were identified and validated by secondary screening. Sequence analyses showed that these encoded nine distinct antigens (Table 1). Interestingly three antigens, ODF2, CEP110 (previously known as CEP1) and CEP250 (previously known as CEP2), are centrosomal proteins while four antigens, TCEB3 (also called Elongin A), RBPJ (previously called RBPSUH), RIF1 and ZBTB44, have (or are postulated to have, in the case of ZBTB44) roles associated with transcription. C14orf93 and ZBTB44 have not previously been characterised, as we did not find any existing publications specifically describing these molecules.

**Table 1:** Sequence and functional information for antigens identified in this study.

Antigen	Frequency	NCBI accession	Location	SEREX database antigen ID (and reactivity-shown in bold font)*	IgG/IgM	Putative/known function
**TCEB3**	1	NM_003198	1p36.1	NGO-Co-21 (Colon cancer);NY-SAR-20 (NK/T-cell malignant sarcoma)	IgG	General transcriptional elongation factor for RNA polymerase II
**BECN1**	2	NM_003766	17q21	-	IgG	Autophagy; possible tumour suppressor
**c14orf93**	2	NM_021944	14q11.2	-	IgG	N-terminal signal peptide, possibly secreted
**ODF2**	1	NM_002540	9q34.11	HOM-Ts-PMR2-69 (Rhematoid arthritis **(2/19 arthritis; 0/12 healthy controls)**;MO-TES-301 (Colon cancer)	IgM	Component of sperm tail cytoskeleton; possible component of centrosomal scaffold in somatic cells
**CEP110**	1	NM_007018NM_153437	9q33-q34	9; MO-TES-250 (Colon cancer);MO-TES-258 (Colon cancer);MO-TES-385 (Colon cancer);NGO-St-64 (Stomach cancer **(1/13 stomach cancer; 1/16 healthy controls))**	IgG	Colocalises with CEP250 in the pericentriolar material of the centrosome; role in centrosome duplication
**CEP250**	1	NM_007186 NM_007018	20q11.22-q12	10; NGO-Br-69 (Breast cancer **(6/31 breast cancer; 1/30 healthy controls))**	IgG	Core centrosomal protein; substrate for NEK2 kinase
**ZBTB44**	3	NM_014155	11q24.3	NGO-Pr-87 (Prostate cancer)	IgG	Contains BTB domain and C_2_H_2_ Zn fingers; possible transcriptional repressor; interacts in TGF-β signalling pathway
**RIF1**	1	NM_018151	2q23.3	-	IgM	S-phase checkpoint, mediated by ATM
**RBPJ**	7	NM_005349 NM_015874NM_203283NM_203284	4p15.2	BRC-Co-5 (Colon cancer);MO-CO-1018 (Colon cancer);MO-CO-126 (Colon cancer);MO-CO-226 (Colon cancer);MO-TES-120 (Colon cancer);Mz19-54b (Melanoma);NGO-Pr-38 (Prostate cancer);NGO-St-1 (Stomach cancer **(7/13 stomach cancer; 6/16 healthy controls)**);NY-BR-8 (breast cancer);NY-CO-79 (Colon cancer);NY-REN -30 (Renal cancer);NY-TLU-65 (Lung cancer)	IgG	Transcription repressor mediating Notch signalling pathway; required for T-cell development by controlling T- versus B-cell fate pathway in lymphoid progenitors

*From the SEREX database (www2.licr.org/CancerImmunomeDB) and PubMed (www.ncbi.nlm.nih.gov/). Reactivity of antigens when known is shown in **bold** font.

*C14orf93* maps to 14q11.2, which is a translocation hotspot in T-cell lymphomas because the alpha T-cell receptor genes map to this locus. The C14orf93 protein sequence is highly conserved across species, having 87% identity between human and murine proteins, which may indicate that this protein performs a functionally important biological role. There are no obvious functional domains predicted in the c14orf93 protein, although a possible cysteine-rich domain was identified and a potential nuclear localisation signal (RRKR) at aa 298 leads to a PSORTII prediction that it might encode a nuclear protein. The greatest similarity to another human protein was to the N-terminal region of KANK4, a protein with a KN motif and ankyrin repeat domains which has a role in forming actin stress fibers [33].

ZBTB44 is exceptionally highly conserved across species, having 96% identity between the human and mouse proteins and 87% identity between human and *Xenopus*. ZBTB44 has N-terminal homology to BACH1, and also exhibits homology to the proto-oncogene Pokemon/ZBTB7A and the BTB protein KLHL12. ZBTB44 has an N-terminal BTB/POZ domain (aa 22–128) that may mediate homo- or hetero-dimerisation [34], four C-terminal C2H2 zinc fingers (aa 399–421, 427–449, 455–479, 487–511) and a nuclear localisation signal, suggesting a likely role in regulating gene expression as a transcription factor.

### Frequency of immunological recognition by lymphoma patients and healthy individuals

**Figure 1:**
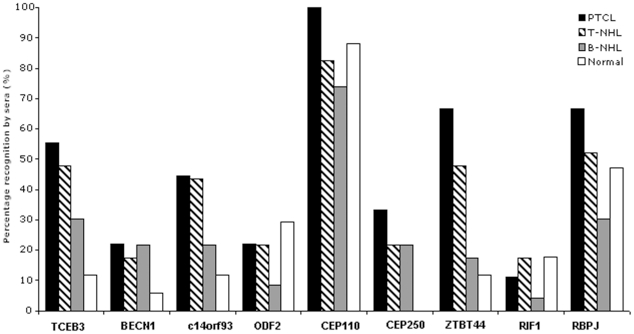
Immunoreactivity of the PTCL, NOS-associated antigens with sera from patients with PTCL, NOS (n = 9), T-NHL (n = 15), B-NHL (n = 32) or healthy controls (n = 17).

To assess the specificity and frequency of the humoural immune responses to these antigens, tertiary screening was performed using 69 sera from nine patients with PTCL, NOS, 15 patients with other T-cell/NK malignancies, 28 patients with B-cell malignancies and 17 normal controls (Table 2). CEP250 was the only antigen that was recognised specifically by patients' but not control sera, showing reactivity with 33% of PTCL, NOS and 23% of B- and T-NHL sera (Figure 1). Four antigens, TCEB3, BECN1, c14orf93, and ZBTB44, showed more frequent recognition by lymphoma patients' sera. Of these, only BECN1 was recognised equally frequently by patients with B- and T-cell malignancies, the remaining antigens being more frequently recognised by sera from patients with T-cell malignancies. ZBTB44 exhibited the highest frequency of patient specific recognition and was recognised by antibodies in sera from 66% of PTCL, NOS patients and only 12% of control sera.

**Table 2:** Reactivity of antibodies present in the sera of lymphoma patients and normal control samples with the PTCL, NOS antigens.

Antigen	PTCL, NOS (n = 9)	ALCL, ALK+* (n = 11)	CTCL (n = 2)	NKT (n = 1)	T-ALL (n = 1)	FL (n = 5)	MCL (n = 5)	DLBCL (n = 5)	HL (n = 5)	MM (n = 3)	BL (n = 2)	MZL (n = 1)	MALT (n = 1)	IC (n = 1)	Cont. (n = 17)
**TCEB3**	5	4	2	0	0	1	3	1	0	1	0	1	0	0	2
**BECN1**	2	1	1	0	0	0	2	2	1	0	0	1	0	0	1
**C14orf93**	4	4	2	0	0	0	2	0	2	1	0	1	1	0	2
**ODF2**	2	3	0	0	0	1	1	0	1	0	0	0	0	0	5
**CEP110**	9	7	2	1	1	4	5	3	5	2	1	1	0	1	15
**CEP250**	3	2	0	0	0	0	2	1	3	0	1	0	0	1	0
**ZBTB44**	6	3	2	0	0	1	0	1	0	1	0	1	0	0	2
**RIF1**	1	2	1	0	0	0	0	0	0	1	0	0	0	0	3
**RBPJ**	6	3	2	1	0	1	1	1	1	2	1	1	0	0	8

Numbers denote the number of sera from that disease type tested that reacted positively at least once in the triplicate analyses, Cont. = normal.

*ALCL, ALK+, anaplastic large cell lymphoma (ALK+); CTCL, cutaneous T cell lymphoma; NKT, natural killer T-cell lymphoma; T-ALL, T cell acute lymphoblastic leukaemia; FL, follicular lymphoma; MCL, mantle cell lymphoma; HL, Hodgkin lymphoma; MM, multiple myeloma; BL, Burkitt lymphoma; MZL, mantle zone lymphoma; MALT, extranodal marginal zone lymphoma of mucosa-associated lymphoid tissue lymphoma; IC, immunocytic lymphoma.

### Analysis of mRNA expression of the PTCL, NOS-associated antigens

**Figure 2:**
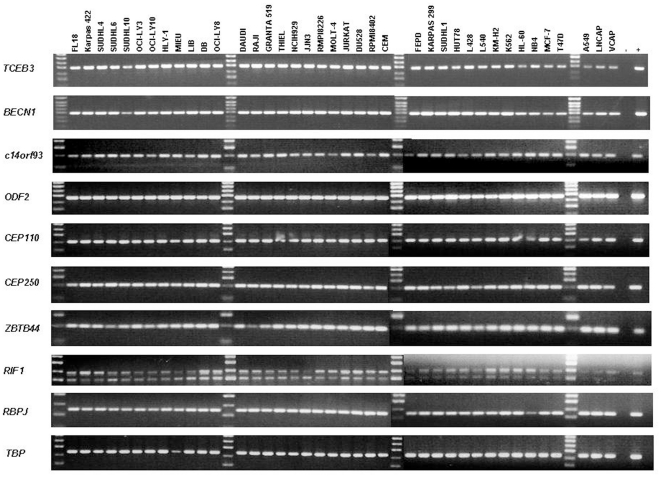
RT-PCR analysis of mRNA expression levels of genes encoding PTCL, NOS-associated antigens in cancer cell lines. −, no reverse transcriptase negative control; +, positive control testis cDNA. *TBP* was included as a positive control for the quality of the cDNA.

**Figure 3:**
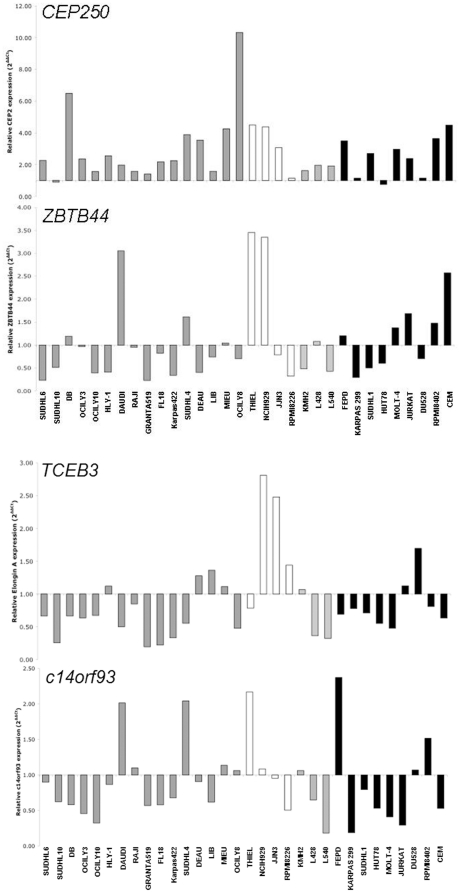
Quantitative real time PCR analysis of genes encoding PTCL, NOS-associated antigens in cancer cell lines. Black bars represent T-cell lines, pale grey are Classical Hodgkin Lyphoma (CHL), white are myeloma and dark grey are B-NHL. Expression was normalised to fractionated normal CD19^+^ B cells for B-cell derived cell lines or fractionated normal CD3^+^ T cells for T-cell derived cell lines.

The mRNA encoding each of the PTCL, NOS-associated antigens was ubiquitously detected in a panel of 39 cancer cell lines using RT-PCR (Figure 2). Two isoforms of *RIF1* were detected and these showed differential relative expression between cell lines. Expression of the four genes encoding antigens that were immunologically recognised by more than 30% of PTCL, NOS patients and less frequently recognised by control sera (TCEB3, c14orf93, CEP250 and ZBTB44) was also characterised using quantitative real time PCR (Figure 3). *CEP250* expression was widely upregulated in lymphoma and myeloma cell lines, when compared to levels in normal fractionated control cells. *ZBTB44* and *c14orf93* were differentially expressed, with *ZBTB44* showing highest expression levels in one B-NHL and two myeloma cell lines and most frequent upregulation (5/9) in cell lines derived from T-cell malignancies. In contrast, *TCEB3* was generally down regulated in most cell lines, with the notable exception being particularly high level expression in three of four myeloma cell lines.

**Figure 4:**
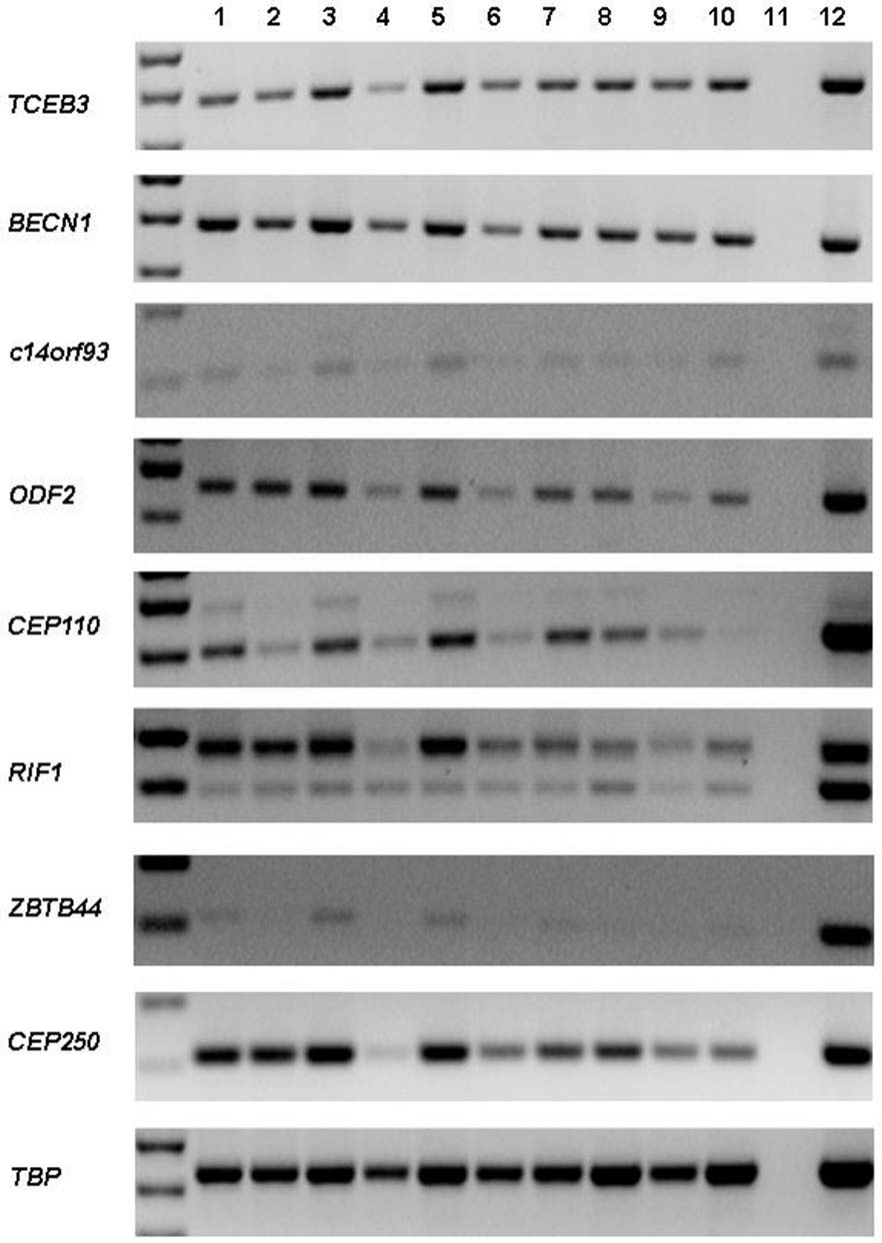
RT-PCR analysis of PTCL, NOS SEREX antigens in fractionated peripheral blood cells. 1, resting CD4^+^T cells; 2, activated CD4^+^ T cells; 3, resting CD8^+^ T cells; 4, activated CD8^+^ T cells; 5, resting CD19^+^ B cells; 6, activated CD19^+^ B cells; 7, resting CD14^+^ monocytes; 8, activated monocytes; 9, mononuclear B and T cells; 10, positive control placenta tissue cDNA; 11, no reverse transcriptase negative control; 12, positive control Thiel cDNA.

The expression of each gene was also investigated by RT-PCR in a panel of fractionated resting and activated peripheral blood cells (Figure 4). *TCEB3*, *BECN1* and *c14orf93* were expressed at comparable levels in both lymphocytes and monocytes, while the remaining genes were preferentially expressed in both B and T lymphocytes. There was an indication that many of the genes showed reduced expression in response to cell activation. This varied in the different T-cell populations for example CD4^+^ T-cell activation had no affect on the expression of *CEP250*, *RIF1*, *ODF2* or *TCEB3*, while all four genes appeared to be expressed at lower levels in activated when compared to resting CD8^+^ T cells. Although these cDNA panels were supplied as semi-quantitative, via normalisation to a range of house keeping genes, there was differing expression of the *TBP* gene used as a loading control. Thus, the expression of the four genes encoding antigens that were immunologically recognised by more than 30% of PTCL, NOS patients (TCEB3, c14orf93, CEP250 and ZBTB44) and less frequently recognised by control sera was also investigated in these samples using quantitative real time PCR (Figure S1). The expression of *c14orf93* was unaffected by activation in CD14^+^ monocytes or CD8^+^ T cells, but was reduced in activated CD19^+^ B cells and activated CD4^+^ T cells. In contrast, *ZBTB44* expression was decreased in activated CD4^+^, CD8^+^ and CD14^+^ cells but was slightly increased in activated CD19^+^ B cells. All four genes demonstrated variable responses to activation suggesting that the regulation of these genes is affected by the cell lineage.

The expression of these genes was also investigated in seven other normal human tissues (Figure S2). *RIF1* was also included to determine whether the two isoforms were differentially expressed amongst normal tissues. All four genes were expressed in non-malignant tissues from brain, colon, ovary, placenta, testis, thymus and leukocytes. Brain and leukocytes expressed relatively low amounts of the smaller *RIF1* isoform when compared to other normal tissues.

### Analysis of protein expression of the PTCL, NOS-associated antigens

**Figure 5:**
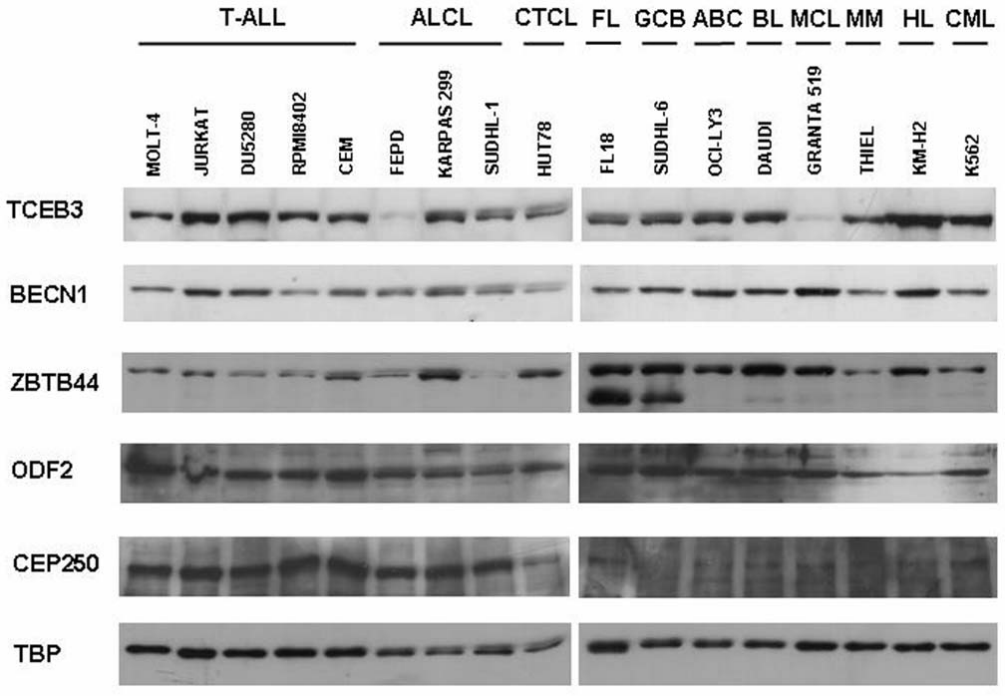
Western blot analysis of antibodies to PTCL, NOS SEREX antigens against selected whole cell RIPA extracts. T-ALL, T cell acute lymphoblastic leukaemia; ALCL, ALK+; CTCL, cutaneous T cell lymphoma; FL, follicular lymphoma; GCB, germinal centre-derived diffuse large B cell lymphoma; ABC, activated B cell-derived diffuse large B cell lymphoma; BL, Burkitt lymphoma; MCL, mantle cell lymphoma; HL, Hodgkin lymphoma; CML, chronic myelogenous leukaemia.

Antibodies were commercially available to five of the nine PTCL, NOS SEREX antigens. All the antibodies detected a protein with a molecular weight consistent with that predicted for the antigen. Consistent with mRNA expression data, all of the antigens were widely expressed amongst cell lines derived from haematological malignancies (Figure 5). Particularly interesting was the differential expression observed for TCEB3, BECN1 and ZBTB44 in both B and T-cell derived cell lines. CEP250 appeared to be more highly expressed in T-cell malignancies. The anti-ZBTB44 antibody detected an additional lower molecular weight protein in the two germinal centre-derived NHL cell lines, this is most likely to represent the detection of an additional ZBTB44 isoform that is expressed in these cells.

## Discussion

Using the SEREX technique we have identified nine antigens that are immunologically recognised by autoantibodies from patients with PTCL, NOS. Four antigens, ODF2, CEP110, RIF1 and RBPJ are also frequently recognised by antibodies in sera taken from healthy control individuals. These represent autoantigens whose immunological recognition is not specifically associated with the presence of lymphoma. Our analysis of the mRNA expression of the remaining five genes, which are preferentially recognised by sera from lymphoma patients, suggests that all are widely expressed in normal tissues. Thus these antigens are unlikely to represent good candidates for lymphoma vaccination, as there may be unwanted side effects on normal somatic tissues. However, this does not exclude the possibility that the five antigens preferentially recognised by sera from patients (TCEB3, BECN1, CEP250, c14orf93 and ZBTB44) may have a role in the pathobiology of lymphoma. Two of the antigens were previously uncharacterised, and with the exception of BECN1 (see below), none have been previously studied in the context of lymphoma.

Six of the identified antigens have been shown to be immunologically recognized by cancer patient sera in previous SEREX studies (Table 1), although none of them in T-cell lymphoma SEREX studies [23], [24], [25], [26], or indeed a more recent study that used proteomic analysis to identify T-cell lymphoma-associated antigens [35]. This is not perhaps surprising as the current study is the first to identify PTCL, NOS-associated SEREX antigens, whereas the other studies investigated sera from cutaneous T-cell lymphoma patients.

CEP250 is a centrosomal protein and was the only antigen specifically recognised by sera from lymphoma patients. At the mRNA level, *CEP250* expression was upregulated in cell lines from haematological malignancies when compared to normal lymphocytes, and there also appeared to be more CEP250 protein in T-cell derived cell lines. The use of antibody interference to study CEP250 (also known as c-Nap1) function caused centrosome splitting, indicating that this antigen may have a role in mediating centriole-centriole cohesion [36]. Centrosome aberrations have been commonly identified in NHL and it has been suggested that these may contribute to the acquisition of chromosomal instability [37]. Furthermore, many oncogenes and tumour suppressor genes control centrosome duplication and function, and thus it has been suggested that centrosomes might prove to be effective cancer targets [38]. Interestingly, chromosomal instability and supernumerary centrosomes have been identified as precursor defects in a mouse model of T-cell lymphoma, which suggests they may have a role in lymphomagenesis [39]. However, although a causal link has now been identified between centrosome dysfunction and tumorigenesis in flies, there is considerable ongoing debate as to whether the mechanism is via genomic instability or deregulation of asymmetric cell division [40].

TCEB3 is a component of the transcription elongation factor B (SIII) complex that regulates the rate of transcription by RNA polymerase II [41]. Loss of *Tceb3* in mice generates an embryonic lethal phenotype and mouse embryonic fibroblasts from these embryos display increased apoptosis and senescence-like growth defects [42]. Also of interest is the observation that RNA polymerase II bypass of oxidative damage, which may contribute to transcriptional mutagenesis and tumour progression, is regulated by transcription elongation factors [43].

*BECN1* is a haplosufficient tumour suppressor gene and *Beclin+/−* mice suffer from a high incidence of spontaneous tumours [44]. This phenotype reflects reduced levels of autophagy, which is the process by which cellular proteins and organelles can be used to generate metabolic precursors to maintain cell growth under nutrient deprivation. At the protein level it appeared that there was less BECN1 expression in the T-cell derived cell lines when compared to the B-cell lines. Significantly, Th2 cells become more resistant to growth factor withdrawal-induced cell death when autophagy is blocked via silencing BECN1 expression [45]. It has also been reported that the failure to sustain metabolism via autophagy may cause genomic instability and promote tumorigenesis, thus the loss of a survival pathway may paradoxically enhance tumour growth [46]. Interestingly, low BECN1 expression was found to be an independent indicator of poor prognostic outcome (PFS and OS) in a recent study of 65 extranodal NKT-cell lymphoma cases [47].

Of particular interest for future investigation are the two previously uncharacterised genes, *c14orf93* and *ZBTB44*, both of which are differentially expressed in lymphoma-derived cell lines. Both antigens are more frequently immunologically recognised by sera from patients with T-cell malignancies and the high sequence conservation across species suggests that they may have an evolutionarily-conserved function. Sequence analysis of the predicted protein products provides little information as to the likely role of c14orf93, but identifies ZBTB44 as a zinc finger and BTB/POZ domain protein. The proteins that share greatest sequence similarity to ZBTB44 have roles in cancer: Bach1 acts as a negative regulator of p53 and connects oxygen metabolism and cellular senescence [48], while Pokemon/ZBTB7A is overexpressed in multiple cancers and its expression is required for the oncogenic transformation of mouse embryonic fibroblasts [49]. Yeast-two-hybrid studies have identified binding of ZBTB44 to components of the Smad signalling pathway, which is regulated by members of the TGFβ superfamily and can be disrupted in cancer [50]. As a number of BTB-containing zinc finger proteins are also implicated in thymocyte development and T-cell function [51], ZBTB44 may potentially play a role in normal or neoplastic T cell development at the transcriptional level, possibly as part of a signalling effector mechanism.

In summary, we have identified a panel of antigens that are immunologically recognised *in vivo* by patients with PTCL, NOS. Several have not previously been studied in lymphoma patients and two antigens were previously uncharacterized. Future studies are needed to determine whether these novel tumor antigens play a role in the pathogenesis of PTCL.

## Supporting Information

Figure S1 **Quantitative real time PCR analysis of genes encoding PTCL, NOS-associated antigens in resting and activated mononuclear cells.** Black bars represent B cells, pale grey are T cells, dark grey are CD27-positive or CD27-negative mononuclear cells and white are monocytes. Expression was normalised to fractionated pooled normal CD19^+^ B-cells (B cells, CD27^+^ and CD14^+^ cells) or CD3^+^ T-cells (T-cells).
(TIF)

Figure S2 **RT-PCR analysis of mRNA expression levels of PTCL, NOS-associated antigens in normal tissues.** −ve, no reverse transcriptase negative control; +ve, positive control testis cDNA. TBP was included as a positive control for the quality of the cDNA.
(TIF)

Table S1 **Oligonucleotides and Taqman assays used for RT-PCR/qRT-PCR.**
(DOCX)

Table S2 **Clinical details of PTCL, NOS patients whose sera were used for library screening using the SEREX technique.**
(DOCX)
