# Supplementary figures and images for: Identification and Characterization of Peripheral T-Cell Lymphoma-Associated SEREX Antigens
Source: PLoS One. 2011 Aug 22;6(8):e23916. doi: 10.1371/journal.pone.0023916 (PMC3161784; doi:10.1371/journal.pone.0023916)

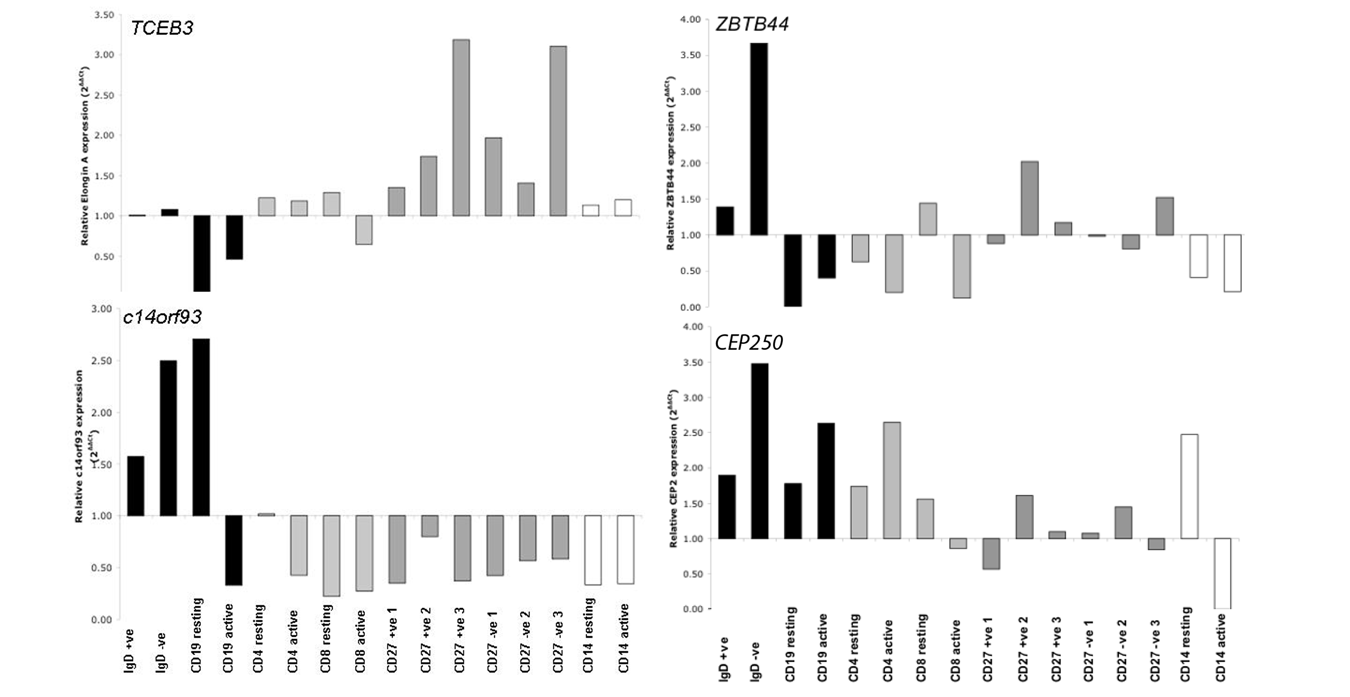

Supplement: Figure S1 — Quantitative real time PCR analysis of genes encoding PTCL, NOS-associated antigens in resting and activated mononuclear cells. Black bars represent B cells, pale grey are T cells, dark grey are CD27-positive or CD27-negative mononuclear cells and white are monocytes. Expression was normalised to fractionated pooled normal CD19+ B-cells (B cells, CD27+ and CD14+ cells) or CD3+ T-cells (T-cells). (TIF) [file pone.0023916.s001.tif]

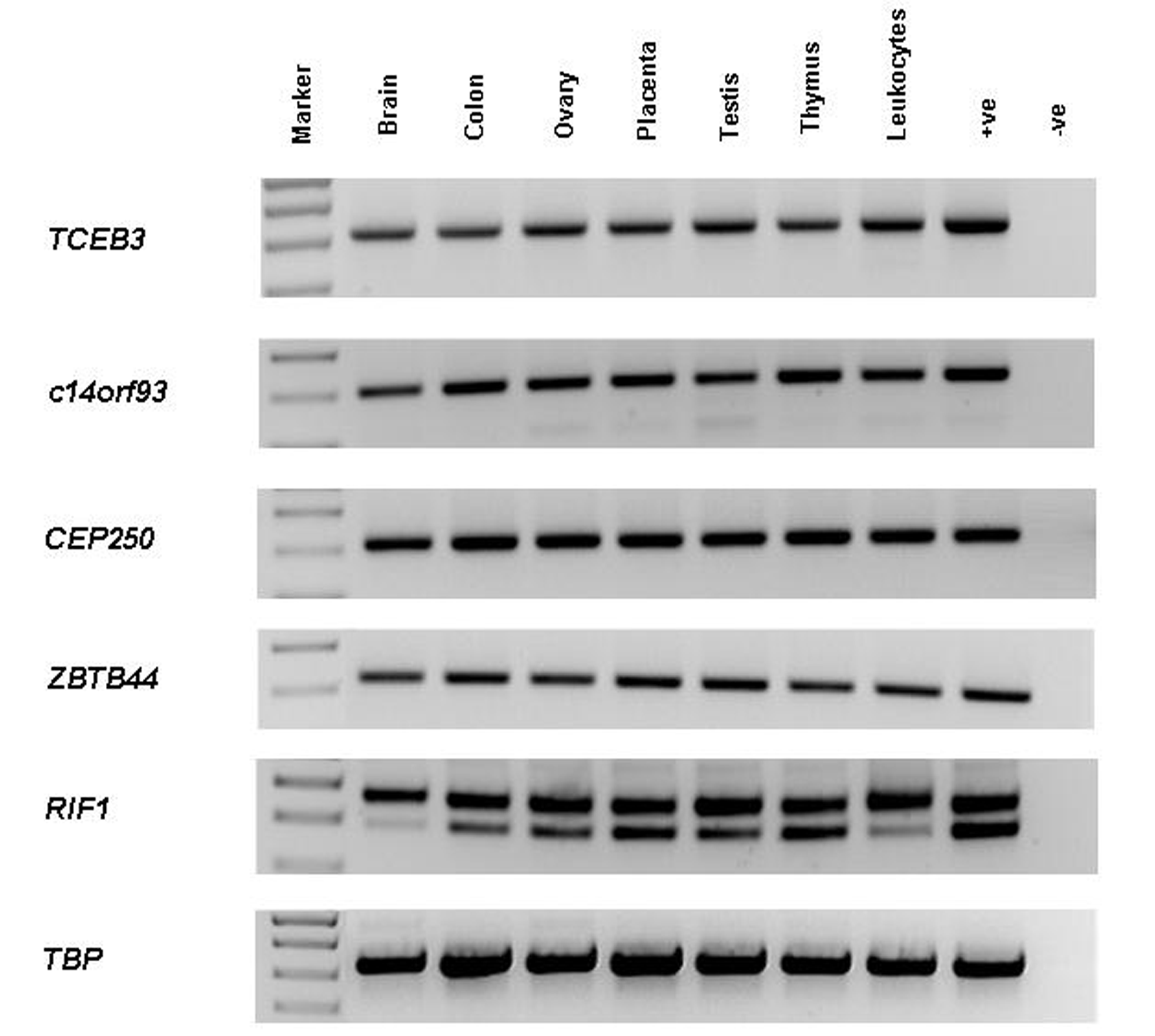

Supplement: Figure S2 — RT-PCR analysis of mRNA expression levels of PTCL, NOS-associated antigens in normal tissues. −ve, no reverse transcriptase negative control; +ve, positive control testis cDNA. TBP was included as a positive control for the quality of the cDNA. (TIF) [file pone.0023916.s002.tif]
