# Supplementary material for: Identification and Characterization of Peripheral T-Cell Lymphoma-Associated SEREX Antigens
Source: PLoS One. 2011 Aug 22;6(8):e23916. doi: 10.1371/journal.pone.0023916 (PMC3161784; doi:10.1371/journal.pone.0023916)
Supplement: Table S1 — Oligonucleotides and Taqman assays used for RT-PCR/qRT-PCR. (DOCX) [file pone.0023916.s003.docx]

**Supplementary Table S1**. Oligonucleotides and Taqman assays used for RT-PCR/qRT-PCR.

| **Gene** | **Primer sequence (5’-3’)** | **PCR**  **(bp)** | **Taqman assay ID** | **Taqman probe sequence (5’-3’)** |
| --- | --- | --- | --- | --- |
| ***TCEB3*** | F:ACCCGGACCCTAAGAAGCTA  R:ACAAGATGGAGGGGATTGAA | 491 | Hs00162605_m1 | AGACATTCTTGCGGAGACTGGGGTT |
| ***BECN1*** | F:GTCACTGGGGACCTTTTTGA  R:CTGTCCACTGTGCCAGATGT | 492 | N/A | N/A |
| ***c14orf93*** | F:AGGTGGTGATTGAGGAGCTG  R:GCATAAGCAGGGACCAGAAC | 202 | Hs00222580_m1 | GACCTTCGGATGCCGGAAGGTCCTT |
| ***ODF2*** | F:GATACCATCGGGAAGCTGAA  R:GCCTCCACTTCTGCCTTATG | 461 | N/A | N/A |
| ***CEP110*** | F:GGACAGGCAGTTAGGGCATA  R:AGCACTGCCACCTGAGTCTT | 405 | N/A | N/A |
| ***RIF1*** | F:CTGTGTCTGGTCTCCTTTGG  R:GGGTAAACACTTTCTGTTGG | 375 | N/A | N/A |
| ***ZBTB44*** | F:GAAGAAGATGTCCGGGTCAA  R:AGTGTAGGCAAGCCCTCAGA | 209 | Hs00274052_m1 | GTCCACAGTCTTCCTCTATAGGCTC |
| ***CEP250*** | F:GAGCTACAGCGAATGGAAGC  R:GGCTCTCTCACGCTCACTCT | 210 | Hs00173227_m1 | AGCCACTGGAGGACCAATCCCCCAG |
| ***RBPJ*** | F:TTGCCCACCTCCTTGTGTAT  R:AGGCAATGCATAAGTCAGCA | 347 | N/A | N/A |
| ***TBP*** | F:CCACAGCTCTTCCACTCACA  R:CACAGCTCCCCACCATATTC | 433 | 4310891E | N/A |
| ***GAPDH*** | F:GAGTCAACGGATTTGGTCGT  R:TGTGGTCATGAGTCCTTCCA | 512 | N/A | N/A |
