# Supplementary material for: Identification and Characterization of Peripheral T-Cell Lymphoma-Associated SEREX Antigens
Source: PLoS One. 2011 Aug 22;6(8):e23916. doi: 10.1371/journal.pone.0023916 (PMC3161784; doi:10.1371/journal.pone.0023916)
Supplement: Table S2 — Clinical details of PTCL, NOS patients whose sera were used for library screening using the SEREX technique. (DOCX) [file pone.0023916.s004.docx]

**Supplementary Table S2.** Clinical details of PTCL, NOS patients whose sera were used for library screening using the SEREX technique.

| **Patient ID** | **Sex** | **Age at diagnosis** | **Stage** | **IPI** | **Treatment** | **Response** |
| --- | --- | --- | --- | --- | --- | --- |
| PerT001 | F | 58 | N/A | N/A | N/A | N/A |
| PerT002 | F | 59 | I | 2 | CHOPx6 | CR |
| PerT003 | M | 79 | IV | 5 | Prednisolone + supportive case | Progressive disease |
| PerT004 | M | 67 | IV | 3 | FMD x1, 1/2 CHOP+rituximab x3 | Progressive disease |
